# *O*-GlcNAc-Mediated Regulation of Galectin Expression and Secretion in Human Promyelocytic HL-60 Cells Undergoing Neutrophilic Differentiation

**DOI:** 10.3390/biom12121763

**Published:** 2022-11-27

**Authors:** Adam McTague, Rada Tazhitdinova, Alexander V. Timoshenko

**Affiliations:** Department of Biology, The University of Western Ontario, London, ON N6A 5B7, Canada

**Keywords:** cell differentiation, neutrophils, galectins, *O*-GlcNAc, unconventional secretion

## Abstract

In this study, we have tested the hypothesis that the expression and secretion of galectins are driven through mechanisms globally impacted by homeostatic regulation involving the post-translational modification of intracellular proteins with *O*-linked N-acetylglucosamine (*O*-GlcNAc). We showed that neutrophilic differentiation of HL-60 cells induced by all-*trans* retinoic acid (ATRA) and 6-diazo-5-oxo-L-norleucine (DON) was associated with a significant drop of cellular *O*-GlcNAc levels in serum-contained and serum-free cell culture media. Galectin gene and protein expression profiles in HL-60 cells were specifically modified by ATRA and by inhibitors of *O*-GlcNAc cycle enzymes, however overall trends for each drug were similar between cells growing in the presence or absence of serum except for *LGALS9* and *LGALS12*. The secretion of four galectins (-1, -3, -9, and -10) by HL-60 cells in a serum-free medium was stimulated by *O*-GlcNAc-reducing ATRA and DON while *O*-GlcNAc-elevating thiamet G (*O*-GlcNAcase inhibitor) failed to change the basal levels of extracellular galectins. Taken together, these results demonstrate that *O*-GlcNAc homeostasis is essential not only for regulation of galectin expression in cells but also for the secretion of multiple members of this protein family, which can be an important novel aspect of unconventional secretion mechanisms.

## 1. Introduction

Cell differentiation is associated with multiple changes in gene and protein expression profiles, and specific post-translational modification of regulatory proteins. Our recent studies with HL-60 promyeloblasts have demonstrated that neutrophilic differentiation leads to non-uniform changes (stimulation or inhibition) in the expression of specific galectins [1,2,3]. Galectins are soluble β-galactoside-binding proteins with diverse glycan-dependent/independent functions outside and inside cells [4,5,6]. There are 16 galectin genes discovered to date, with 12 expressed in humans [7], and 6 of which are expressed in the acute myeloid leukemia cell line HL-60 [1,8]. Several of these galectins are inducible in HL-60 cells during granulocytic differentiation and all of them are considered as powerful regulators of neutrophil functions such as extravasation, chemotaxis, phagocytosis, and oxidative burst among others [9]. Although galectins are found in circulation in mammals including humans, mice, and cattle [10,11,12,13], molecular mechanisms controlling their secretion remain poorly understood since these proteins lack an ER-targeting signal sequence and therefore use unconventional secretory transport pathways [14,15].

Emerging evidence suggests that localization, trafficking, and secretion of soluble proteins may depend on cellular *O*-GlcNAc homeostasis, which implies the level of intracellular proteins glycosylated with a single sugar *O*-linked *N*-acetylglucosamine (*O*-GlcNAc). *O*-GlcNAcylation is driven by two enzymes: *O*-GlcNAc transferase (OGT) uses UDP-GlcNAc as a substrate and adds the sugar moiety to specific serine or threonine residues while *O*-GlcNAcase (OGA) removes *O*-GlcNAc [16,17]. UDP-GlcNAc is produced by the hexosamine biosynthetic pathway, the rate-limiting step of which is catalyzed by glucosamine fructose-6-phosphate transaminase (GFPT, composed of ubiquitous GFPT1 and less common GFPT2). The global level of *O*-GlcNAc significantly varies between different human tissues and cells [18], increases in response to stress stimuli [19], and decreases during differentiation of many cell lineages [3,20]. Living organisms need to maintain and control *O*-GlcNAc homeostasis since its dysregulation leads to disorders associated with either high or low *O*-GlcNAc levels, which are typical, for instance, in cancer and neurodegenerative diseases, respectively [21]. Secretion of some proteins such as insulin and growth hormones are inhibited by drugs, which elevate the level of *O*-GlcNAcylation in cells [22,23]. Considering that galectin molecules have sites for *O*-GlcNAcylation, we have hypothesized that the secretion of galectins in cells might be controlled by *O*-GlcNAc-dependent mechanisms. In particular, we have proposed that high levels of *O*-GlcNAc promote intracellular accumulation of galectins in stem cells while low levels of *O*-GlcNAc promote galectin secretion and cell differentiation [20,24]. By this time, this hypothesis has been tested and supported for *O*-GlcNAc-controlled secretion of galectin-3 in two different models such as nutrient-sensing responses of HeLa cells and mouse embryonic fibroblasts [25] and mouse extraembryonic endoderm differentiation [26], however other experimental confirmations are warranted considering a complex network of galectins in cells.

To develop an adequate model for studying *O*-GlcNAc-dependent expression and secretion of galectins, we revisited and assessed neutrophilic differentiation of acute promyelocytic leukemia HL-60 cells in serum-contained and serum/galectin-free media. Here, we show that the hallmarks of all-*trans* retinoic acid (ATRA)-induced neutrophilic differentiation such as lobular morphology of nuclei [27,28], upregulation of genetic biomarkers *NCF1* (Neutrophil Cytosolic Factor 1) and *NCF2* [2], and respiratory burst in response to phorbol 12-myristate 13-acetate (PMA) [29] are maintained in both media in association with a significant drop of *O*-GlcNAcylated proteins in cells. Moreover, the expression patterns of individual galectins undergo similar changes in both media in response to inhibitors of *O*-GlcNAc cycle enzymes, although the magnitude has been variable. Based on the serum/galectin-free cell culture model, we provide evidence in support of our *O*-GlcNAc homeostasis hypothesis for secretion of multiple galectins (galectin-1, galectin-3, galectin-9, and galectin-10) by HL-60 cells. We showed that the secretion of these galectins was increased when cells were exposed to ATRA and GFPT inhibitor 6-diazo-5-oxo-L-norleucine (DON), both inducing cellular differentiation and decreasing *O*-GlcNAcylation of intracellular proteins. In comparison, inhibition of OGA by thiamet G and subsequent elevation of *O*-GlcNAc did not change the basal levels of extracellular galectins. Taken together, our findings demonstrate that *O*-GlcNAc homeostasis is essential for both the regulation of galectin synthesis in cells and the secretion of multiple members of this protein family, providing novel insights into molecular mechanisms of unconventional secretion and cell biology of galectins.

## 2. Materials and Methods

### 2.1. Reagents and Solutions

All-*trans*-retinoic-acid (ATRA) (R2625), 6-diazo-5-oxo-L-norleucine (DON) (D2141), horseradish peroxidase (P-8250), thiamet G (TG) (SML0244), Dulbecco’s Phosphate Buffered Saline (DPBS) with MgCl_2_ and CaCl_2_ (D8862), and scopoletin (S2500) were purchased from Sigma-Aldrich Canada (Oakville, ON, Canada). DPBS without MgCl_2_ and CaCl_2_ (311-425-CL), 100× ITS Universal Culture Supplements (315-081-QL), and Iscove’s Modification of Dulbecco’s Modified Eagle Medium (IMDM) (319-105-CL) were purchased from Wisent Bio Products (Saint-Jean-Baptiste, QC, Canada). EDTA (EB0185), 100× Mammalian Protease Inhibitor Cocktail (BS386), 2× RIPA Buffer IIII with EDTA and EGTA (pH 7.4) (RB4477), sodium azide (S2002), bovine serum albumin (BSA) (AD0023), and sodium orthovanadate (SB0869) were purchased from BioBasic (Markham, ON, Canada). Nonfat dry milk blotting-grade blocker (1706404) was purchased from Bio-Rad (Mississauga, ON, Canada). VECTASHIELD Vibrance Mounting Medium with DAPI (H-1800) was purchased from Vector Laboratories (Burlingame, CA, USA). Phorbol 12-myristate 13-acetate (PMA) (PMA168) was purchased from BioShop (Toronto, ON, Canada). Ambion TRIzol^®^ (15596018) was purchased from Life Technologies (Toronto, ON, Canada). Fetal Bovine Serum (FBS) (12484-028, Gibco™), and SYBR™ Safe DNA Gel Stain (S33102, Invitrogen, Eugen, OR, USA) were purchased from ThermoFisher Scientific (Mississauga, ON, Canada). Acryl/Bis™ 29:1 ULTRA PURE 40% (*w*/*v*) Solution (0311) was purchased from VWR Life Science (Mississauga, ON, Canada). Molecular biology grade agarose Froggarose LE (A87) was purchased from FroggaBio (Concord, ON, Canada). OGT inhibitor 2-acetamido-1,3,4,6-tetra-O-acetyl-2-deoxy-5-thio-α-D-glucopyranose (Ac-5SGlcNAc or AC) was synthesized as described elsewhere [30] and kindly provided by Dr. David Vocadlo as per Material Transfer Agreement between Simon Fraser University and the University of Western Ontario.

### 2.2. Cell Culture and Treatments

Human acute promyelocytic leukemia HL-60 cells (ATCC^®^ CCL-240^TM^) were cultured as a suspension in IMDM without antibiotics and supplemented with either 10% FBS (IMDM-FBS) or ITS (5 μg/mL human insulin, 5 μg/mL human transferrin, 5 ng/L selenous acid) (IMDM-ITS) in a humidified incubator at 37 °C, 5% CO_2_. Cells were sub-cultured twice weekly in 100 × 20 mm suspension culture dishes (83.3902.500, Sarstedt, Montreal, QC, Canada) and concentration was kept below 10^6^ cells/mL. Cell viability was determined using the trypan blue (0.4%) exclusion test and was not less than 90% except where indicated.

To induce neutrophilic differentiation, HL-60 cells were grown in either 60 × 15 mm or 100 × 20 mm suspension culture dishes from Sarstedt and treated with 1 μM ATRA for 72 h as introduced elsewhere [31], unless indicated otherwise. To change global *O*-GlcNAcylation, the cells were treated with 10 μM TG (OGA inhibitor) [3], 25 μM AC (OGT inhibitor) [3], or 12.5 μM DON (GFPT inhibitor) [32] for up to 72 h using either a single dose or daily supplementation every 24 h.

### 2.3. RNA Isolation, cDNA Synthesis, and PCR Gene Expression Assays

Following treatments, cells were centrifuged for 5 min at 300× *g*, washed once with ice-cold DPBS without Mg^2+^ and Ca^2+^, and the total mRNA was isolated from cell pellets using TRIzol^®^ reagent according to manufacturer’s protocol. The mRNA samples were dissolved in sterile nuclease-free water and their purity and quantity were assessed with the Thermo Scientific™ Nanodrop™ 2000c spectrophotometer (Wilmington, DE, USA) considering an A_260/280_ ratio of 1.8–2.0 as a minimum threshold for use in later assays. cDNA was synthesized from 500 ng of mRNA using the High-Capacity cDNA reverse transcription Kit from Applied Biosystems (4368813, ThermoFisher Scientific) as per the manufacturer’s protocol.

All primers for polymerase chain reaction (PCR) assays were synthesized by BioCorp UWO OligoFactory (Western University, Department of Biochemistry, London, ON, Canada) and verified by nucleotide BLAST (Table A1, Appendix A). Primers were dissolved in nuclease-free water at a concentration of 100 μM and used to prepare primer master mixes containing 10 μM of both forward and reverse primers. The end-point and quantitative PCR assays were performed in T100 Thermo Cycler (Bio-Rad) or CFX Connect Real-Time PCR Detection System (Bio-Rad), respectively, as previously described [1,2,3]. The reaction volume of PCR samples in both cases was 20 μL and contained 1 μM forward/reverse primers, 0.5 μL of undiluted cDNA, and either Taq FroggaMix (FroggaBio, FBTAQM) or SsoAdvanced Universal SYBR^®^ Green Supermix (Bio-Rad, 1725274). PCR amplicons were separated on a 2% agarose gel prepared in TAE buffer (20 mM Tris, 40 mM acetic acid, 1.2 mM EDTA) containing SYBR^®^ Safe and imaged using a Molecular Imager^®^ Gel Doc™ XR+ (Bio-Rad) with ImageLab software, version 6.1 (Bio-Rad). A 2-step cycling regime was used for real-time qPCR following polymerase activation and cDNA denaturation for 30 s at 95 °C, 40 cycles of denaturation (5 s, 95 °C) and annealing (25 s, 60–65 °C). Annealing temperatures varied based on primer pair and specificity of qPCR amplification was verified by the presence of a single melt peak at a specific temperature for each amplicon (Table A1). Relative transcript levels were calculated by the Livak method (2^−ΔΔCT^) using β-actin (*ACTB*) as a reference gene [33].

### 2.4. Western and Immunodot Blotting

To prepare cell pellet lysates, cell culture suspensions were centrifuged at 300× *g* for 5 min, rinsed twice with ice-cold DPBS and lysed in 300 μL of RIPA buffer (50 mM Tris-HCl, pH 7.4, 0.1% sodium dodecyl sulfate, 0.5% *w*/*v* sodium deoxycholate, 5 mM EDTA, 1 mM EGTA and 150 mM NaCl) supplemented with a protease inhibitor cocktail (1 mM phenylmethylsulfonyl fluoride, 100 μM Na_3_VO_4_, 10 μM bestatin, 14 μM E-64, 10 μM leupeptin, 0.8 μM aprotinin and 15 μM pepstatin A). The cell lysates were incubated on ice for 10 min and centrifuged at 12,000× *g* for 15 min at 4 °C. Total protein concentration was quantified using the DC^TM^ Protein Assay Kit II (5000112) from Bio-Rad and BSA as a standard by measuring absorbance at 655 nm in a model 3550 Microplate Reader (Bio-Rad).

For Western blot analysis, samples of cell lysates were mixed with a 4X SDS sample loading buffer with β-mercaptoethanol, boiled for 5 min, loaded on 4% polyacrylamide stacking gel (25 μg protein per lane), and electrophoretically separated at 100 V by SDS-PAGE in 8% (for *O*-GlcNAc detection) or 12% (for galectin detection) polyacrylamide resolving gels. The proteins were transferred from the gel to PVDF membrane (0.2 μm pore size, Sigma-Aldrich, 03010040001) at a constant voltage of 20 V at 4 °C overnight using wet transfer in a 25 mM Tris, 190 mM glycine, 20% (*v*/*v*) methanol (pH 8.3) buffer. Afterwards, the membrane was blocked with a 3% non-fat dry milk reconstituted in TBS buffer (20 mM Tris-HCl, pH 7.5, 150 mM NaCl) supplemented with 0.05% Tween 20 (TBST) for 60 min at room temperature, washed three times with TBST and incubated overnight at 4 °C with an appropriate primary antibody solution (15 mL) in TBST supplemented with 5% BSA and 0.05% sodium azide. The membrane was washed again 3 times with TBST and then incubated with either goat-anti-mouse or goat-anti-rabbit IgG-HRP conjugated secondary antibody diluted 1:10,000 in a 5% non-fat dry milk reconstituted in TBST for 1 h at room temperature with light agitation. Lastly, the TBST-rinsed membrane was evenly covered with 1.5 mL of Immobilon Classico Western HRP substrate (Sigma-Aldrich, WBLUC500) and imaged using a ChemiDoc XRS system with Quantity One Software, version 4.6.6 (Bio-Rad). Western blots were quantified using ImageLab software, version 6.1 (Bio-Rad) and band intensities were normalized to β-actin as a loading control.

To analyze global levels of selected galectins and *O*-GlcNAcylated proteins, the cell lysates were immobilized onto nitrocellulose membrane (0.2 μm pore size, GE Healthcare 1060006) using a Bio-Dot^®^ Microfiltration apparatus (Bio-Rad) as previously described [3,34,35]. Briefly, the nitrocellulose membrane was prewetted in TBS and placed into the apparatus. Each well was loaded with 200 μl of whole cell protein extract (20 μg/mL in DPBS) and proteins were transferred to the membrane by gravity filtration for 90 min. Blocking, antibody incubation, and imaging steps for immunodot blots were performed as described above for Western blots. Densitometry and quantification of immunodot blots was performed using ImageLab software, version 6.1 (Bio-Rad) with the subsequent normalization of dot intensities to the average intensity of control samples. 

The primary and secondary antibodies were obtained from Santa Cruz Biotechnology (Dallas, TX, USA), Abcam Inc (Toronto, ON, Canada), and ThermoFisher Scientific (Mississauga, ON, Canada) (Table A2, Appendix A).

### 2.5. Enzyme-Linked Immunosorbent Assay (ELISA)

Cell suspensions were centrifuged at 300× *g* for 5 min and the supernatants (8–10 mL) were collected and frozen at −80 °C to use later for analyses of extracellular proteins. Concentrations of secreted galectin-1, galectin-3, and galectin-9 were measured using ELISA kits from Abcam (ab260053, ab269555, and ab213786) as described by the manufacturer. For all three kits, cell culture supernatant samples were diluted 4-fold in the supplied sample diluent that was found to fall within the range of concentrations that correspond to the standard curve. Absorbance at 450 nm was measured using a Model 3550 Microplate Reader (Bio-Rad).

### 2.6. Scopoletin Assay to Measure PMA-Induced H_2_O_2_ Production

H_2_O_2_ production by HL-60 cells was measured by scopoletin/peroxidase fluorescence assay using an AMINCO-Bowman Series 2 luminescence spectrometer (SLM AMINCO, Urbana, IL, USA) as described earlier [2,36] with minor modifications. Briefly, cell culture suspensions were centrifuged for 5 min at 300× *g* and the cell pellets were washed twice with and resuspended in DPBS containing Mg^2+^ and Ca^2+^ to yield a concentration of 6 × 10^5^ cells/mL. Scopoletin (500 nM) and horseradish peroxidase (20 μg/mL) were added to 2 mL of the cell suspension, which was prewarmed for 5 min in a cuvette of the fluorimeter at 37 °C before adding 1 μM of PMA. A decrease in fluorescence of scopoletin was monitored at 460 nm (excitation at 350 nm) and the maximal slope of the recorded traces was calculated in RStudio (version 4.0.5) to quantify the rate of H_2_O_2_ generation by cells [2].

### 2.7. Nuclear Staining, Fluorescence Microscopy, and Nuclear Morphology

**Figure 1:**
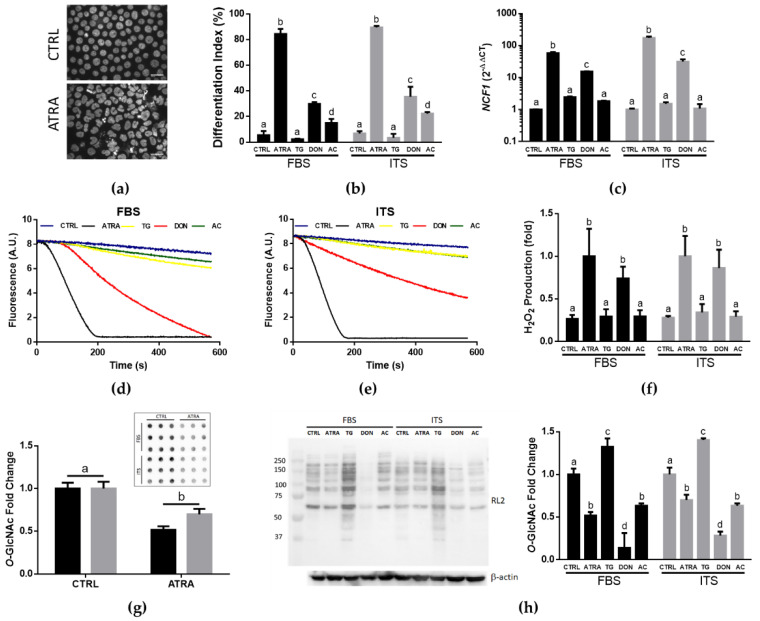
Effects of ATRA and *O*-GlcNAc cycle inhibitors on the hallmarks of HL-60 differentiation in serum-free and serum-contained cell culture media. HL-60 cells were cultured in IMDM supplemented with either FBS or ITS and treated with ATRA (1 μM) or *O*-GlcNAc inhibitors (10 μM TG, 12.5 μM DON, or 25 μM AC) for 72 h with daily drug supplementation. (**a**) Reference images of DAPI-stained HL-60 cells demonstrating ovoid and non-ovoid (indented, lobulated, or multilobed) nuclear morphology of undifferentiated (CTRL) and differentiated (ATRA) cells in IMDM-ITS medium, respectively. Scale bars, 20 μm; (**b**) Differentiation indices of HL-60 cells based on the nuclear morphology (percentage of cells with lobular nuclei); (**c**) Changes in the expression of neutrophilic differentiation marker *NCF1*; (**d**,**e**) Representative time traces of PMA-induced oxidation of scopoletin (0.5 μM, 20 μg/mL HRP) by HL-60 cells (6 × 10^5^ cells/mL) grown in IMDM medium supplemented with FBS or ITS, respectively; (**f**) Quantification of PMA-induced H_2_O_2_ generation by HL-60 cells based on the traces showed in (**d**,**e**) (*n* = 4–5); (**g**) Immunodot blot (insert) obtained using RL2 antibody and the ensuing quantification of global *O*-GlcNAc levels in undifferentiated (CTRL) and differentiated (ATRA) HL-60 cells grown in media supplemented with FBS (black bars) or ITS (grey bars); (**h**) Western blot of *O*-GlcNAcylated proteins detected using RL2 antibody and the ensuing quantification of integral intensity for each lane are shown. *O*-GlcNAc fold changes were calculated as a ratio of the integral intensities of all bands to β-actin as a loading control with the subsequent normalization to the average of untreated cells (CTRL). Significant differences (*p* < 0.05) between treatments are labeled by different letters as per one-way ANOVA followed by a Tukey’s multiple comparisons test for each media. The results are presented as means ± SD, *n* = 3.

To prepare slides for nuclear staining, HL-60 cells were diluted to a concentration of 5 × 10^5^ cells/mL in 200 μL and centrifuged onto glass slides for 5 min at 500 rpm using the Shandon Cytospin 2 centrifuge (Shandon Southern Products, Cheshire, England) [1]. The attached cells were fixed with chilled methanol for 5 min and staining of nuclei was achieved by mounting the slides with DAPI-contained VECTASHIELD Vibrance Mounting Medium. An AxioImager A1 fluorescence microscope (Carl Zeiss, Toronto, ON, Canada) equipped with DAPI filter cube was used to view the slides. The images (Figure 1a) were taken with a high-resolution monochrome XCD-X700 CCD camera (Sony of Canada Ltd., Toronto, ON, Canada) using Northern Eclipse 8.0 software from Empix Imaging (Mississauga, ON, Canada) and used to calculate the differentiation index, i.e., the percentage of nuclei with non-ovoid (indented, lobulated, or multilobed) morphology representing differentiated cells versus nuclei with ovoid/rounded morphology representing undifferentiated cells [27,28]. At least three randomly selected areas were imaged on each slide, the numbers of ovoid (N_1_) and non-ovoid (N_2_) nuclei were counted using ImageJ (11–46 cells per image), and the differentiation index was calculated as N_2_/(N_1_ + N_2_) × 100%.

### 2.8. Statistical Analysis

One-way and two-way ANOVA followed by Tukey’s multiple comparisons test, Student’s *t*-test, and Pearson’s correlation test were performed using GraphPad Prism, version 6.01 for Windows (GraphPad Software, La Jolla, CA, USA). The ‘cocor’ R package was used to analyze statistically significant differences between Pearson’s correlations as described earlier [37]. All experiments were conducted using a minimum of three biological replicates. Data were presented as means ± SD and differences between means were considered significant at *p* < 0.05.

## 3. Results

### 3.1. Effects of ATRA and Inhibitors of O-GlcNAc Cycle Enzymes on Neutrophilic Differentiation of HL-60 Cells in Serum-Contained and Serum-Free Cell Culture Media

**Figure A1:**
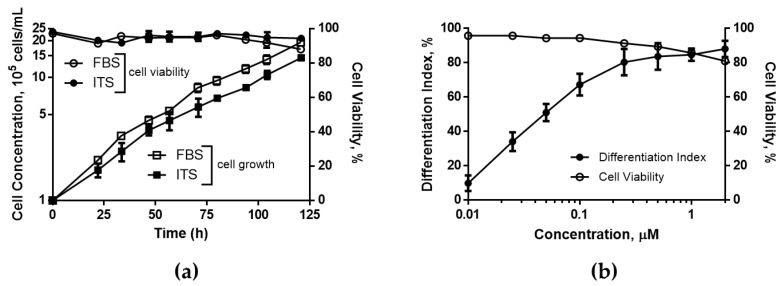
HL-60 cells growth and differentiation in serum-free medium: (**a**) Cells in IMDM with FBS (10%) or ITS (5 μg/mL human insulin, 5 μg/mL human transferrin, 5 ng/L selenous acid) were grown in 60 mm suspension culture dishes at an initial concentration of 10^5^ cells/mL for 5 days in a humidified incubator set to 37 °C and 5% CO_2_. Cell concentrations and viability were recorded every 12 h. No differences in the population doubling time (22.3 ± 2.7 h versus 23.4 ± 4.9 h) and cell viability were noticed between IMDM-FBS and IMDM-ITS media; (**b**) Dose-dependent effect of ATRA on viability and differentiation of HL-60 cells in IMDM-ITS serum-free medium. Cell viability and differentiation were assessed using trypan blue test and nuclear morphology index, respectively, as described in Materials and Methods. Data are presented as means ± SD, *n* = 3.

**Figure A2:**
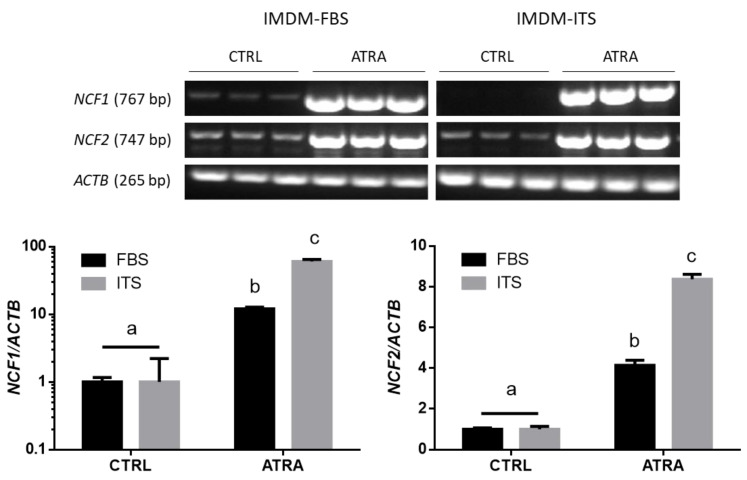
ATRA-induced upregulation of *NCF1* and *NCF2* genes encoding essential p47-phox and p67-phox subunits of the plasma membrane NADPH oxidase in HL-60 cells. Cells (4 × 10^5^ cells/mL) were treated with 1 μM ATRA for 72 h to induce neutrophilic differentiation. The end-point RT-PCR was performed with 3 biological replicates in each case as described in Material and Methods using the primers specified in Table A1. The PCR products were separated on 2% agarose gel and the expression of *NCF1* and *NCF2* was quantified based on the intensity of bands normalized to ACTB as a house-keeping gene. Data are presented as means ± SD, *n* = 3. Different letters are used to label means which are significantly different (*p* < 0.05) based on two-way ANOVA followed by Tukey’s HSD test.

As mammalian serum contains galectins [10,11,12,13], we sought initially to compare the growth and differentiation of HL-60 cells in serum-contained (IMDM-FBS) and serum-free (IMDM-ITS) media to develop an exogenous galectin-free model for subsequent studying galectin secretion in cell culture. The population doubling time of HL-60 cells growing in IMDM-FBS and IMDM-ITS cell culture media was statistically the same, 22.3 ± 2.7 h versus 23.4 ± 4.9 h (Figure A1, Appendix A). Next, to analyze neutrophilic differentiation of HL-60 cells, we selected ATRA as a common and potent inducer of cell differentiation [8,31] with well-established mechanisms of activation through nuclear retinoic acid/retinoid X receptors [38]. Previous studies showed that the important hallmarks of ATRA-induced differentiation of HL-60 cells in a regular serum-contained cell culture medium are lobular/non-ovoid morphology of nuclei [27,28], overexpression of cytosolic components of plasma membrane NADPH oxidase [29], and reduction of *O*-GlcNAc levels in differentiated cells [32] and we addressed these aspects of HL-60 differentiation in our model. As per nuclear morphology assay in IMDM-FBS medium, ATRA induced efficient differentiation of HL-60 cells in a dose-dependent manner reaching a plateau at 1 μM over 72 h treatment (Figure A1, Appendix A). This concentration was chosen for all further experiments, which showed no differences in the changes of the nuclear morphology differentiation index between IMDM-FBS and IMDM-ITS media (Figure 1b). The same patterns were also observed for ATRA-induced upregulation of *NCF1* and *NCF2* genes encoding essential p47-phox and p67-phox subunits of plasma membrane NADPH oxidase (Figure 1c and Figure A2, Appendix A) and the relevant PMA-induced generation of H_2_O_2_ as a functional response of differentiated cells (Figure 1d–f). These changes were concomitant with the same reduction of *O*-GlcNAc levels in both media as assessed by immunodot and Western blotting (Figure 1g,h). Thus, serum/galectin-free IMDM-ITS provides a proper medium to study ATRA-induced neutrophilic differentiation of HL-60 cells.

To determine how critical *O*-GlcNAc homeostasis is in the context of neutrophilic differentiation, we compared the ATRA-induced changes with the effects of biochemical drugs, which are known to elevate or reduce *O*-GlcNAc in cells by inhibiting OGA (TG, 10 μM), OGT (AC, 25 μM) and GFPT (DON, 12.5 μM), respectively [3,32]. As such, *O*-GlcNAc repressor DON readily mimicked the effects of ATRA resulting in significant elevation of differentiation index based on nuclear morphology (Figure 1b), upregulation of *NCF1* (Figure 1c), and acquisition of the ability to generate H_2_O_2_ in response to PMA (Figure 1d–f). Surprisingly, AC was only efficient in inducing morphological changes of nuclei without affecting other hallmarks of neutrophilic differentiation at the dose used. In comparison, *O*-GlcNAc enhancer TG failed to change all these parameters preventing any signs of neutrophilic differentiation and keeping HL-60 cells in their progenitor state (Figure 1b–f). Altogether, these results confirmed that low *O*-GlcNAc is required for neutrophilic differentiation while high *O*-GlcNAc is a feature of stem-like, undifferentiated cells. 

### 3.2. Galectin Expression Profiles Depend on O-GlcNAc Homeostatic Changes in HL-60 Cells

**Figure 2:**
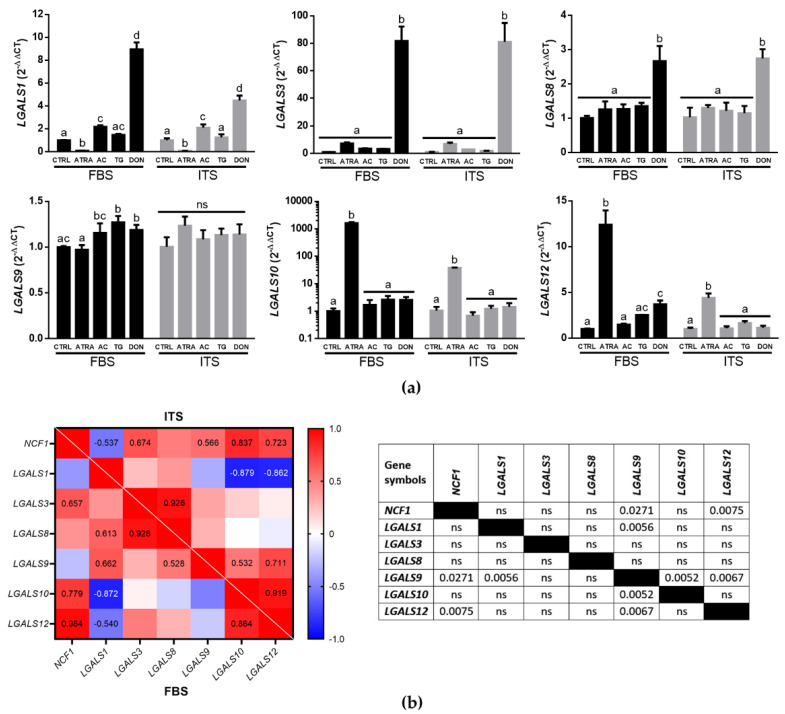
Sensitivity of galectin gene expression to disruptions in *O*-GlcNAc homeostasis: (**a**) Changes in the expression of six galectin genes (*LGALS1*, *LGALS3*, *LGALS8*, *LGALS9*, *LGALS10*, and *LGALS12)* in HL-60 cells grown in IMDM medium supplemented with either FBS or ITS and treated with *O*-GlcNAc-modulating drugs as specified in Figure 1. Significant differences (*p* < 0.05) between treatments are labeled by different letters as per one-way ANOVA followed by a Tukey’s multiple comparisons test for each media. The results are presented as means ± SD, *n* = 3–5; (**b**) Pairwise correlation analysis of gene expression in HL-60 cells: left, heatmap showing pairwise correlation patterns between expression of genes (log2-transformed values) encoding galectins and cell differentiation marker *NCF1*. Significant (*p* < 0.05) Pearson’s correlation coefficients are reported for each pairwise comparison while blanks cells indicate that the correlation is not significant; right, comparison of pairwise Pearson’s correlations between IMDM-FBS and IMDM-ITS samples using the ‘cocor’ R package, significant *p* values are shown, ns—not significant, *n* = 15 for each gene (3 biological replicates for each treatment including control).

Having proved the role of *O*-GlcNAc in neutrophilic differentiation of HL-60 cells, we next questioned the expression of galectins in HL-60 cells as molecules which have been proposed to function in an *O*-GlcNAc-dependent manner [20,24]. The expression of six galectin genes was assessed using RT-qPCR in HL-60 cells grown in different cell culture media (IMDM-FBS and IMDM-ITS) and treated with either 1 μM ATRA, 10 μM TG, 25 μM AC, or 12.5 μM DON for 72 h with daily drug supplementation to maintain consistent high or low *O*-GlcNAc levels in cells (Figure 2a). Two-way ANOVA revealed significant changes in the expression of all examined genes including *LGALS1* (F_4,20_ = 537.2, *p* < 0.0001), *LGALS3* (F_4,20_ = 242.6, *p* < 0.0001), *LGALS8* (F_4,20_ = 51.60, *p* < 0.0001), *LGALS9* (F_4,20_ = 4.822, *p* < 0.01), *LGALS10* (F_4,26_ = 706.9, *p* < 0.0001), and *LGALS12* (F_4,20_ = 185.0, *p* < 0.0001) between treatments. No interactions were observed between the type of cell culture medium and treatments for the expression of *LGALS3* (F_4,20_ = 0.016, *p* = 0.999), *LGALS8* (F_4,20_ = 0.361, *p* = 0.833), and *LGALS9* (F_4,20_ = 5.169, *p* = 0.05) while the significant interactions were noticed for *LGALS1* (F_4,20_ = 77.15, *p* < 0.0001), *LGALS10* (F_4,26_ = 646.2, *p* < 0.0001), and *LGALS12* (F_4,20_ = 54.15, *p* < 0.0001). However, Tukey’s multiple comparisons HSD test revealed common patterns in the expression of galectin genes depending on the treatments versus control: TG treatment (high *O*-GlcNAc) did not change the expression of all six galectin genes except for an upregulation of *LGALS9* in serum-contained medium; DON (low *O*-GlcNAc) induced significant upregulation of *LGALS1*, *LGALS3*, and *LGALS8* in all media, *LGALS12* in IMDM-FBS only, and had no effects on *LGALS9* and *LGALS10*; AC (moderate low *O*-GlcNAc) induced significant upregulation of only *LGALS1* and failed to change the expression of other galectin genes; ATRA (moderate low *O*-GlcNAc) significantly inhibited the expression of *LGALS1*, stimulated the expression of *LGALS10* and *LGALS12*, had diverse effects on *LGALS9* (stimulation in serum-free medium with no effect in serum-contained medium), and did not change the expression of *LGALS3* and *LGALS8* (Figure 2a).

Given the complexity of changes in the expression of galectin genes between IMDM-FBS and IMDM-ITS media, we split the data into two sets and performed a pairwise correlation analysis between genes of interest and *O*-GlcNAc to reveal potential associations in their regulation. The heatmaps of correlation coefficients for both IMDM-FBS and IMDM-ITS media demonstrated overall similar patterns of multiple significant positive and negative correlations including 10 cases in IMDM-FBS dataset and 11 cases in IMDM-ITS dataset, 7 of each were overlapped (Figure 2b). Further, the comparison of correlations between these two datasets showed no significant differences in most cases, except for several correlations accompanying *LGALS9* and *LGALS12*. Significant differences between cell culture media were noticed for *LGALS9* correlations in association with *NCF1*, *LGALS1*, *LGALS10*, and *LGALS12* as well as for *LGALS12/NCF1* pair (Figure 2b). Remarkably, differences between IMDM-FBS and IMDM-ITS pairwise correlations for *LGALS12/NCF1* pair were significant only for log_2_-transformed values (Figure 2b) and not detected when comparing original gene expression levels (not shown). In spite of these variations, the correlation analysis showed high level of consistency in *O*-GlcNAc/galectin gene expression associations between two cell culture models.

**Figure 3:**
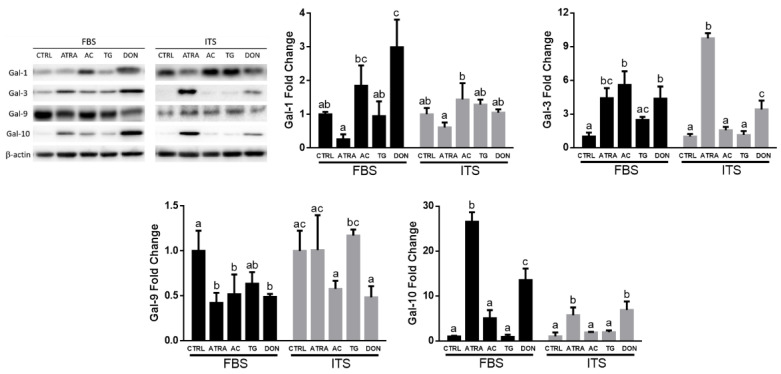
Effects of ATRA and *O*-GlcNAc cycle inhibitors on the intracellular levels of galectins. HL-60 cells were grown in IMDM medium supplemented with either FBS or ITS and treated with *O*-GlcNAc-modulating drugs as specified in Figure 1. Representative Western blots and the ensuing quantification of galectin-1, galectin-3, galectin-9, and galectin-10 levels in HL-60 cells are shown. The fold changes were calculated as a ratio of galectin band intensities to β-actin as a loading control with the subsequent normalization to the average of untreated cells (CTRL). Significant differences (*p* < 0.05) between treatments are labeled by different letters as per one-way ANOVA followed by a Tukey’s multiple comparisons test for each media. The results are presented as means ± SD, *n* = 3–5.

Since the gene expression is not always consistent with protein abundance, next we used Western blotting to quantify the intracellular levels of galectin-1, galectin-3, galectin-9, and galectin-10 in HL-60 cells treated with drugs that modulate *O*-GlcNAc status (Figure 3). Galectin-12 was not included in this part of our study because none of the commercially available antibodies were efficient in detecting galectin-12 in HL-60 cells. Two-way ANOVA revealed significant changes in the protein levels of all examined galectins between treatments including galectin-1 (F_4,20_ = 14.18, *p* < 0.001), galectin-3 (F_4,21_ = 73.61, *p* < 0.001), galectin-9 (F_4,20_ = 8.144, *p* < 0.001), and galectin-10 (F_4,20_ = 116.8, *p* < 0.001). Significant interactions between the type of cell culture medium and treatments were noticed for all galectins including galectin-1 (F_4,20_ = 8.633, *p* < 0.001), galectin-3 (F_4,21_ = 38.74, *p* <0.001), and galectin-9 (F_4,20_ = 3.734, *p* < 0.05), and galectin-10 (F_4,20_ = 53.10, *p* < 0.001). However, overall common trends were noticed in both media including ATRA- and DON-induced upregulation of galectin-3 and galectin-10 as well as AC-induced upregulation of galectin-1, although with different magnitudes. None of the galectins at a protein level demonstrated a decrease in response to all treatments except for galectin-9 in ATRA/AC/DON-treated cells in IMDM-FBS. TG treatments (high *O*-GlcNAc) did not change the protein levels of all tested galectins in our model. Thus, the global *O*-GlcNAc-associated changes in galectin profiles of HL-60 cells were similar between IMDM-FBS and IMDM-ITS media. 

### 3.3. Effects of ATRA and O-GlcNAc Cycle Enzyme Inhibitors on the Secretion of Galectins from HL-60 Cells

**Figure 4:**
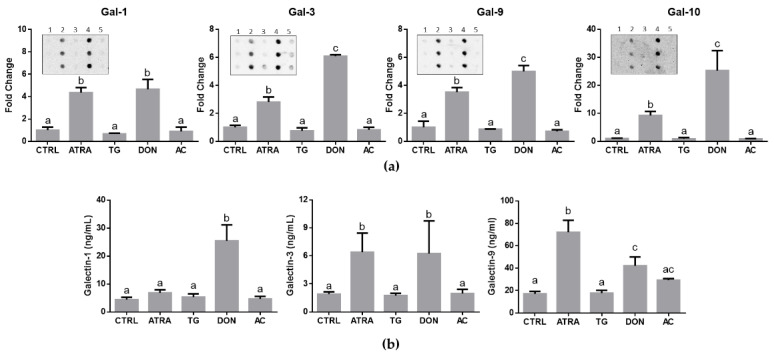
Effects of ATRA and *O*-GlcNAc cycle inhibitors on the secretion of galectins from HL-60 cells. The cells were grown for 72 h in IMDM-ITS, treated with *O*-GlcNAc-modulating drugs as specified in Figure 1, and cell supernatants were collected for analysis of galectin levels as described in Materials and Methods: (**a**) Immunodot blots (inserts; 1, 2, 3, 4, 5 labels correspond to CTRL, ATRA, TG, DON, and AC samples, respectively) for galectin-1, galectin-3, galectin-9, and galectin-10 in serum-free cell supernatants and their quantification by densitometric analysis with normalization to control samples; (**b**) Accumulation of secreted galectin-1, galectin-3, and galectin-9 in serum-free culture medium at 72 h, as detected by ELISA. Significant differences (*p* < 0.05) between treatments are labeled by different letters as per one-way ANOVA followed by a Tukey’s multiple comparisons test. The results are presented as means ± SD, *n* = 3–5.

To conduct this part of our study, we used the established advantage of serum/galectin-free IMDM-ITS medium to assess the *O*-GlcNAc-dependent secretion of endogenous galectins by HL-60 cells. Two complementary immunotechniques were applied to evaluate the levels of secreted galectins in supernatants of HL-60 cells treated for 3 days with ATRA (1 μM), DON (12.5 μM), AC (25 μM) and TG (10 μM): immunodot blots and ELISA. As per one-way ANOVA test, immunodot blots revealed highly significant changes in the secretion of four tested galectins including galectin-1 (F_4,10_ = 50.47, *p* < 0.0001), galectin-3 (F_4,10_ = 313.1, *p* < 0.0001), galectin-9 (F_4,10_ = 111.4, *p* < 0.0001), and galectin-10 (F_4,10_ = 39.80, *p* < 0.0001). More specifically, the secretion of all galectins was significantly increased by cells with low *O*-GlcNAc (treatments with ATRA and DON) in comparison with high *O*-GlcNAc (treatment with TG and control) while no changes were noticed in response to AC (Figure 4a). 

To supplement these findings, ELISA was used to determine the concentrations of secreted galectins-1, -3, and -9 in cell supernatants. The basal levels of these galectins in a 3-day culture of HL-60 cells (normalized to 10^6^ cells) were 4.46 ± 0.81 ng/mL (galectin-1), 1.89 ± 0.26 ng/mL (galectin-3), and 17.14 ± 2.13 ng/mL (galectin-9). Similar to immunodot blots, there were significant changes in the concentrations of galectin-1 (F_4,10_ = 33.53, *p* < 0.0001), galectin-3 (F_4,24_ = 10.12, *p* < 0.0001), and galectin-9 (F_4,10_ = 41.16, *p* < 0.0001) within a cohort of untreated and treated cells (Figure 4b). Treatments with DON increased the secretion of all three galectins by 5-fold, 3-fold, and 2-fold for galectins-1, -3, and -9, respectively while ATRA induced the secretion of only galectin-3 and galectin-9 by 2-fold and 4-fold, respectively (Figure 4b). The effects of tested doses of TG and AC on galectin secretion were not significantly different from control in all cases, although there was an increasing trend for galectin-9 secretion by 71% in the presence of AC.

## 4. Discussion

Human acute promyelocytic leukemia HL-60 cells present an excellent model to study the role of galectins and *O*-GlcNAc in processes of neutrophilic differentiation [2,3,8], however the presence of galectins in circulation and consequently in FBS may impose possible restrictions and limitations for unbiased analysis since animal galectins can interfere with functional responses of human cells [39]. In fact, our findings justify that a serum/galectin-free cell culture medium (IMDM supplemented with ITS) provides proper conditions for growth and ATRA-induced differentiation of HL-60 cells similar to serum (10% FBS)-contained cell culture. This similarity was evident for multiple hallmarks of ATRA-induced neutrophilic differentiation including the formation of lobular morphology of nuclei, upregulation of genetic biomarkers *NCF1* and *NCF2* encoding essential p47-phox and p67-phox subunits of plasma membrane NADPH oxidase, respiratory burst of differentiated cells in response to PMA, and a significant drop of *O*-GlcNAcylated proteins in cells detected using RL2 antibody (Figure 1). Moreover, the expression patterns of individual galectins at gene and protein levels demonstrated similar changes and main effects in both media in response to inhibitors of *O*-GlcNAc cycle enzymes despite significant interactions in several cases. These may be not interpretable as crossover interactions [40], instead detecting mostly differences in the magnitude of the dependent variable changes under *O*-GlcNAc homeostasis perturbations. Indeed, pairwise correlation analysis of galectin and *NCF1* gene expression revealed no significant differences between IMDM-FBS and IMDM-ITS media except of *LGALS9* cohort and *LGALS12/NCF1* pair (Figure 2b). The latter two cases suggest that the role and regulation of *LGALS9* and *LGALS12* in neutrophilic differentiation may not be generalized with other galectin genes and additional mechanisms might be considered in the context of effects of uncharacterized serum components versus ITS supplement, which requires future studies. In comparison, our findings suggest that galectin-3 and galectin-10 can be considered as reliable biomarkers of neutrophilic differentiation in both media, which is consistent with previous studies performed in serum-contained media [2,3]. An important novel aspect of our findings is that this notion is supported by not only significant positive correlations between expression of galectin genes and *NCF1* (a marker of granulocytic differentiation) but also by an inverse relationship between intracellular levels of galectin proteins and *O*-GlcNAc, which is commonly high in many undifferentiated and stem cells [3,20,26]. Thus, the presence of serum or exogenous galectins in cell culture medium is not essential for the initiation of ATRA-induced differentiation of HL-60 cells that provides a convenient model to investigate cell-specific mechanisms of subsequent galectin/*O*-GlcNAc regulation including the secretory pathways.

Previous studies showed that *O*-GlcNAcylation of intracellular proteins is a powerful mechanism controlling multiple cellular processes including responses to stress [19], self-renewal of normal and cancer stem cells [41,42,43], and cellular differentiation [20] among others. Differentiation programs of many cell lineages are associated with decreasing *O*-GlcNAc levels, however this concept may not be generalized to all cell types as there are a few exceptions such as adipocytes [44], chondrocytes [45], osteoblasts [46], and corneal epitheliocytes [47] and also there are significant differences between basal levels of *O*-GlcNAc in human tissues [18]. As such, the local tissue-specific *O*-GlcNAc homeostasis might be essential for normal development and functions of cells, tissues, and organs while a disturbance of *O*-GlcNAc homeostasis balance is a feature of neurodegenerative diseases, cancer, and diabetes [21]. Our findings support the differentiation program strategy of those cell lineages which prefer an alternative post-translational modification of regulatory proteins rather than *O*-GlcNAcylation. Indeed, all signs of neutrophilic differentiation of HL-60 cells were readily induced not only by a conventional inducer ATRA but also by DON (an inhibitor of GFPT, rate-limiting enzymes of hexosamine biosynthetic pathway), both of them significantly reduced the level of *O*-GlcNAcylated proteins in cells. These suggest the requirement of low *O*-GlcNAc levels and deficiency of UDP-GlcNAc as a triggering factor for the process of granulocytic differentiation of HL-60 cells, which is consistent with other studies but in serum-contained media [3,32,48]. Although some effects of an OGT inhibitor (AC) were similar to *O*-GlcNAc-lowering ATRA and DON (lobular nuclear morphology, upregulation of *LGALS3* in IMDM-FBS, downregulation of *LGALS9* in IMDM-FBS, and similar trends of galectin-9 secretion detected by ELISA), a single dose of AC used in this study was not sufficient to induce upregulation of *NCF1* and assembly of functionally active NADPH-oxidase complex in response to PMA, which requires further elaboration. Remarkable in this context that other OGT inhibitors such as OSMI-1 and benzyl 2-acetamido-2-deoxy-α-D-galactopyranoside as well as *OGT* knockdown using shRNA were reported to induce characteristic signs of neutrophilic differentiation including the surface expression of CD11b and CD14 markers in HL-60 and OCI-AML3 leukemia cells, albeit with some cytotoxic effects [32]. It would be interesting to explore in the future whether the magnitude of *O*-GlcNAc changes or enzymatic context and off-target effects of pharmacological inhibitors may be responsible for these variations. Even if it would be not sufficient, a drop in *O*-GlcNAc is required for neutrophilic differentiation program since the treatment of HL-60 cells with *O*-GlcNAc-raising OGA inhibitor TG failed to induce changes in the functional response of cells and expression of galectins/*NCF1* unlike ATRA or DON justifying that high *O*-GlcNAc is a feature of undifferentiated HL-60 cells. 

**Figure 5:**
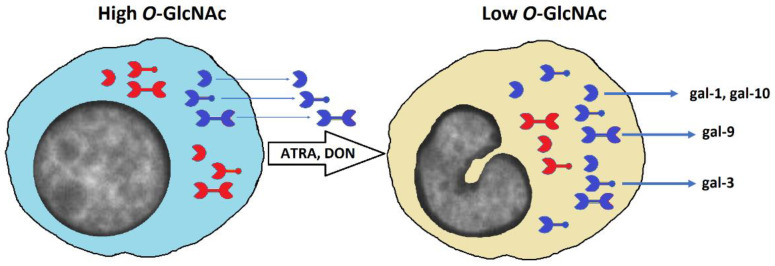
Proposed model of *O*-GlcNAc-mediated expression and secretion of galectins. The model implies that neutrophilic differentiation of HL-60 cells is associated with a decrease of *O*-GlcNAcylated proteins in cells including galectins (resting galectins are in blue and *O*-GlcNAcylated galectins are in red), which results in their secretion. Shape represents the type of galectins: prototype (gal-1, gal-10), chimera type (gal-3), and tandem-repeat type (gal-9). Whereas the secretion of all these galectins is stimulated by a drop in *O*-GlcNAc, changes in intracellular concentration can vary.

Focusing on cell biology of galectins, our findings suggest that galectins can be a target of *O*-GlcNAc-mediated regulation at both gene and protein levels including the secretion of these molecules from cells. Molecular mechanisms of unconventional secretion of galectins, which are synthesized in cytosol, are complex and include possibilities of direct translocation, exosomal and microvesicular trafficking including autophagosomal secretion among others [14,15,49,50]. As such, many galectins (galectin-1, galectin-3, galectin-4, galectin-7, and galectin-10) are detectable in human plasma by mass spectrometry [20] and claimed to have diagnostic value for cancer and cardiovascular diseases [51,52] while others like galectin-12 may accumulate in cytoplasm by binding with lipid droplets [53]. Our *O*-GlcNAc homeostasis hypothesis suggesting that low *O*-GlcNAc microenvironment favors galectin secretion [24] provides a novel direction for understanding the molecular context of this process. This concept has recently been supported by a comprehensive study from the Hanover group, which discovered that galectin-3 can be *O*-GlcNAcylated and reported the following essential findings among others about the secretion of galectin-3 in human and mouse cell models: (1) mouse embryonic fibroblasts from *OGA* knockout mice (high *O*-GlcNAc) secreted less galectin-3 than from wild-type control; (2) shRNA-induced knockdown of *OGA* (high *O*-GlcNAc) inhibited secretion of galectin-3, while OGT inhibitor OSMI-1 (low *O*-GlcNAc) stimulated galectin-3 secretion from HeLa cells; (3) mostly deglycosylated galectin-3 was secreted from HeLa cells; (4) HeLa cells and mouse embryonic fibroblasts secreted more galectin-3 in media without glucose [25]. This has been consistent with our concurrent findings in a model of mouse extraembryonic endoderm differentiation, which revealed an increased secretion of galectin-3 by extraembryonic endoderm XEN cells (low *O*-GlcNAc) in comparison with embryonic stem cells (high *O*-GlcNAc) as well as by embryonic stem cells in response to alloxan, which inhibits *O*-GlcNAcylation [26]. To the best of our knowledge, the current study is the first to address the effects of *O*-GlcNAcylation on secretion of multiple galectins in a model of neutrophilic differentiation of human acute promyelocytic leukemia HL-60 cells. In addition to galectin-3, secretion of three other galectins (galectin-1, galectin-9, and galectin-10) were induced by *O*-GlcNAc-reducing treatments with cell differentiation stimuli, ATRA and DON (Figure 4). All these galectins have predicted sites for *O*-GlcNAcylation [20] and at least galectin-1 was previously reported to be affected by this type of post-translational modification [54]. Therefore, it would be extremely interesting to investigate in the future whether the deglycosylation mechanism described for galectin-3 [25] can be applied to the unconventional secretion of other galectins. The relevant model of galectin expression and secretion that considers our findings with HL-60 cells and potential regulatory role of *O*-GlcNAc is presented in Figure 5. To validate this model, further research with different cell lines is required in the future.

Taken together, we have demonstrated that neutrophilic differentiation of HL-60 cells in both serum-contained and serum-free cell culture media is associated with low *O*-GlcNAc levels and complex remodeling of galectin gene/protein expression profiles, which results in increasing secretion of multiple galectins. A significance of galectin/*O*-GlcNAc relationship and regulation might have more general biological context since this was also demonstrated in animal models of physiological stress such as bird flight migration [34] and ground squirrel hibernation [35]. Delineating the mechanistic interplay between *O*-GlcNAc homeostasis and expression/localization of galectins will help to understand better the cell biological role of these glycan-binding proteins that serve inside and outside the cell as a special type of regulatory molecules possessing no enzymatic activity. 

## Data Availability

The published article includes all data generated or analyzed during this study.

## Appendix A

**Table A1:** PCR primer sequences and characteristics.

Gene Name		Sequence 5′-3′	Size bp	2 Step Cycling	Melting Peak Temperature	PMID Reference
*LGALS1*	F	CCTGGAGAGTGCCTTCGAGTG	220	95 °C (5 s) 60 °C (25 s)	86.5 °C	23108139
	R	CTGCAACACTTCCAGGCTGG				
*LGALS3*	F	CAGAATTGCTTTAGATTTCCAA	108	95 °C (5 s) 60 °C (25 s)	80 °C	18202194
	R	TTATCCAGCTTTGTATTGCAA				
*LGALS8*	F	TGGGGACGGGAAGAGATCAC	172	95 °C (5 s) 62 °C (25 s)	82.5 °C	28365246
	R	TGCCATAAATGCCCAGAGTGTC				
*LGALS9*	F	CTTTCATCACCACCATTCTG	91	95 °C (5 s) 62 °C (25 s)	84 °C	18202194
	R	ATGTGGAACCTCTGAGCACTG				
*LGALS10* *(CLC)*	F	GGATGGCCAAGAATTTGAACTG	82	95 °C (5 s) 62 °C (25 s)	80 °C	28365246
	R	GGTGTAAGAGGATTGGCCATTG				
*LGALS12*	F	TGTGAGCCTGAGGGACCA	111	95 °C (5 s) 65 °C (25 s)	87 °C	18202194
	R	GCTGAGATCAGTTTCTTCTGC				
*NCF1*	F	GTCAGATGAAAGCAAAGCGA	93	95 °C (5 s) 60 °C (25 s)	85.5 °C	23147401
	R	CATAGTTGGGCTCAGGGTCT				
*NCF1 **	F	ACCCAGCCAGCACTATGTGT	767	N/A	N/A	10754283
	R	AGTAGCCTGTGACGTCGTCT				
*NCF2 **	F	CGAGGGAACCAGCTGATAGA	747	N/A	N/A	10754283
	R	CATGGGAACACTGAGCTTCA				
*ACTB*	F	TCAGCAAGCAGGAGTATGACGAG	265	95 °C (5 s) 60 °C (25 s)	84 °C	21029043
	R	ACATTGTGAACTTTGGGGGATG				

Notes: * These primers were used for the end-point PCR.

**Table A2:** Primary and secondary antibodies used for immunodot blot and Western blot assays.

Antigen	Host	Type	Conjugates	Dilution	Source	Catalog #
Galectin-1	Mouse	monoclonal	N/A	1:800	Santa Cruz Biotechnology	sc-166618
Galectin-3	Rabbit	polyclonal	N/A	1:200	Santa Cruz Biotechnology	sc-20157
Galectin-9	Rabbit	monoclonal	N/A	1:1000	Abcam	ab227046
Galectin-10	Rabbit	monoclonal	N/A	1:10,000	Abcam	ab157475
*O*-GlcNAc (RL2)	Mouse	monoclonal	N/A	1:1000	Invitrogen	MA1-072
Mouse IgG (H + L)	Goat	polyclonal	HRP	1:10,000	Invitrogen	A16066
Rabbit IgG (H + L)	Goat	polyclonal	HRP	1:10,000	Invitrogen	A16096
